# First‐Principles Multiscale Modeling of Mechanical Properties in Graphene/Borophene Heterostructures Empowered by Machine‐Learning Interatomic Potentials

**DOI:** 10.1002/adma.202102807

**Published:** 2021-07-23

**Authors:** Bohayra Mortazavi, Mohammad Silani, Evgeny V. Podryabinkin, Timon Rabczuk, Xiaoying Zhuang, Alexander V. Shapeev

**Affiliations:** ^1^ Chair of Computational Science and Simulation Technology Institute of Photonics Department of Mathematics and Physics Leibniz Universität Hannover Appelstraße 11 30167 Hannover Germany; ^2^ Cluster of Excellence PhoenixD (Photonics, Optics, and Engineering–Innovation Across Disciplines) Gottfried Wilhelm Leibniz Universität Hannover 30169 Hannover Germany; ^3^ Department of Mechanical Engineering Isfahan University of Technology Isfahan 84156‐83111 Iran; ^4^ Skolkovo Institute of Science and Technology Skolkovo Innovation Center Nobel St. 3 Moscow 143026 Russia; ^5^ College of Civil Engineering Department of Geotechnical Engineering Tongji University Shanghai 1239 China

**Keywords:** first‐principles calculations, machine learning, mechanical/failure response, multiscale modeling

## Abstract

Density functional theory calculations are robust tools to explore the mechanical properties of pristine structures at their ground state but become exceedingly expensive for large systems at finite temperatures. Classical molecular dynamics (CMD) simulations offer the possibility to study larger systems at elevated temperatures, but they require accurate interatomic potentials. Herein the authors propose the concept of first‐principles multiscale modeling of mechanical properties, where ab initio level of accuracy is hierarchically bridged to explore the mechanical/failure response of macroscopic systems. It is demonstrated that machine‐learning interatomic potentials (MLIPs) fitted to ab initio datasets play a pivotal role in achieving this goal. To practically illustrate this novel possibility, the mechanical/failure response of graphene/borophene coplanar heterostructures is examined. It is shown that MLIPs conveniently outperform popular CMD models for graphene and borophene and they can evaluate the mechanical properties of pristine and heterostructure phases at room temperature. Based on the information provided by the MLIP‐based CMD, continuum models of heterostructures using the finite element method can be constructed. The study highlights that MLIPs were the missing block for conducting first‐principles multiscale modeling, and their employment empowers a straightforward route to bridge ab initio level accuracy and flexibility to explore the mechanical/failure response of nanostructures at continuum scale.

## Graphical abstract

A robust concept of first‐principles multiscale modeling of mechanical properties based on machine‐learning interatomic potentials conveniently trainable over short ab initio datasets is proposed. It is shown that mechanical/failure responses of complex nanostructures at continuum scale and high temperatures can be explored with the precision of sophisticated first‐principles calculations, affordable computational cost, and without the need for empirical data.

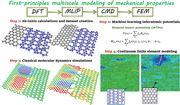

## 1 Introduction

Accurate examination of mechanical responses of nanostructures is vital for the optimal engineering design of nanodevices. In this regard in order to minimize safety issues and failure probabilities during a nanodevice operation, the mechanical properties of each building block and their interactions should be carefully examined. Moreover, as the most realistic scenario, various types of defects exist in the nanomaterials, and their effects on the mechanical properties should also be elaborately investigated. For the conventional bulk materials, uniaxial tensile tests are extensively carried out to examine the mechanical properties of different samples at a wide range of temperatures. When compared with conventional materials, the in‐depth understanding of mechanical properties of nanomaterials is drastically more complicated, and diverse sources of uncertainties related to the measurement technique and samples quality can affect the reliability and reproducibility of estimations. As a rapidly growing field of research, 2D materials with atomic thick structures are gradually entering the daily‐life nanodevices. For materials with only a few nanometers thickness, establishing effective connections between structural information, microstructure, types of defects and environmental effects with the resulting mechanical properties is a very complicated and expensive experimental procedure. Therefore, the development of efficient and accurate numerical modeling approaches to elaborately study the mechanical responses of nanomaterials is highly required to minimize the need for complex, expensive, and time‐consuming experimental endeavors.

First‐principles density functional theory (DFT) simulations are currently extensively employed to examine the mechanical properties of novel materials, with an excellent level of accuracy and reproducibility. The computational cost of common DFT simulations nonetheless scales very fast with the number of atoms, and they are thus limited to studying very small systems with a few hundred or, in extreme cases, a few thousands of atoms. The other major drawback is that DFT simulations of mechanical properties are mostly conducted at ground state, and, thus, the temperature effects are not directly taken into account in the calculations. Atomic vibrations can substantially affect the deformation and failure mechanism and consequently influence the predicted mechanical response. By increasing the temperature, the symmetry of the structures decreases, and larger representative volume elements ought to be considered in the modeling. It is thus clear that the DFT method faces a severe computational cost issue for the assessment of mechanical behavior at high temperatures.

Classical molecular dynamics (CMD) is another popular atomistic‐based simulation approach to explore the mechanical properties of nanostructured systems. The computational cost of CMD simulations scales linearly with the number of atoms, and they are thus considerably more flexible to capture temperature and microstructure effects in the calculations than their DFT counterpart. Unlike the DFT method, CMD estimations nonetheless strongly depend on the accuracy of interatomic potentials. Therefore, CMD models may yield nonphysical and/or inaccurate results depending on the choice of the functional form of empirical interatomic potential or corresponding parameter sets. To better explain this critical bottleneck, one should consider the case of graphene with completely flat and full‐sp^2^ carbon atoms. Despite scattering between different experimental reports, the tensile strength of pristine graphene is expected to be around 130 ± 10 GPa, as experimentally measured by Lee et. al.^[^ ^1^ ^]^ Using the Terosff,^[^ ^2^ ^]^ AIREBO,^[^ ^3^ ^]^ ReaxFF,^[^ ^4^ ^]^ and optimized Tersoff^[^ ^5^ ^]^ original parameter sets, the tensile strength of graphene were predicted to be 200,^[^ ^6^, ^7^ ^]^ 150–250,^[^ ^8^, ^9^ ^]^ 125–138,^[^ ^10^ ^]^ and 158 GPa,^[^ ^11^ ^]^ respectively. All the aforementioned classical interatomic potentials nonetheless predict nonphysical strain hardening at high strain levels. This aforementioned artifact can be removed by the trial and error modification of the potential's cutoff distance and trying to reproduce the experimental results.^[^ ^9^, ^11^ ^]^ It is thus clear that even for the case of graphene, which is the simplest and most‐studied 2D material, the original parameter sets of different interatomic potentials fail to accurately reproduce the mechanical properties. For the case of borophene nanosheets, the 2D forms of boron atoms, CMD methods based on the reactive forcefields not only predict very irregular stress–strain curves,^[^ ^12^ ^]^ but noticeably even fail to reproduce the lattice constants.^[^ ^13^ ^]^ On the other hand, for the majority of novel materials, viable interatomic potentials are not available, and developing a parameter set for a potential function requires a complex fitting procedure. It is therefore conspicuous that compared with DFT counterpart, the CMD models based on the empirical potential functions not only suffer from accuracy issues but also face a flexibility challenge to simulate complex and novel compositions.

**Figure 1:**
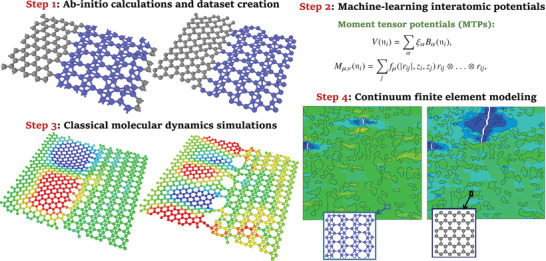
Proposed first‐principles multiscale modeling strategy to simulate the mechanical/failure response of graphene/borophene coplanar heterostructures.

Machine‐learning‐based methods have recently been gaining remarkable attention to address critical challenges in diverse fields, also in materials science.^[^ ^14^, ^15^, ^16^, ^17^, ^18^, ^19^, ^20^, ^21^, ^22^ ^]^ In order to explore the mechanical and structural properties of novel materials and nanostructures,^[^ ^23^, ^24^, ^25^, ^26^, ^27^, ^28^, ^29^ ^]^ machine‐learning interatomic potentials (MLIPs)^[^ ^30^, ^31^, ^32^, ^33^, ^34^ ^]^ show extraordinary capabilities. MLIPs are trained over the first‐principles datasets, and thus they not only exhibit the same order of accuracy but also have the inherent flexibility to study diverse compositions. Since MLIP‐based calculations are conducted using the same platform as that of the common CMD approach, they can be employed to simulate large systems and capture temperature effects. More importantly, MLIPs offer a unique possibility to conduct first‐principles multiscale modeling, in which ab initio level of accuracy can be hierarchically bridged to explore the mechanical/failure response of macroscopic systems. In our earlier study,^[^ ^35^ ^]^ we show that MLIPs can be used to conduct first‐principles multiscale modeling of lattice thermal conductivity. In this work, we step forward and propose the more challenging concept of first‐principles multiscale modeling of mechanical/failure properties. In order to practically show this advanced possibility, we investigate the mechanical properties of coplanar graphene/χ_3_ borophene heterostructures,^[^ ^36^ ^]^ as a novel challenging system, which is beyond the empirical‐based CMD models to be studied reliably. The first‐principles multiscale modeling strategy includes four major steps, which are schematically illustrated in **Figure** **1**. In the first step, ab initio molecular dynamics (AIMD) simulations are conducted over stress‐free and strained monolayers to prepare dataset for passive training. In the second step, an efficient and convenient passive fitting strategy is employed to develop MLIPs for the subsequent CMD calculations. We then conduct MLIP‐based CMD calculations to evaluate the mechanical properties of pristine and heterostructure phases at room temperature. In the final step, on the basis of data acquired by MLIP‐based CMD the mechanical/failure responses of macroscopic heterostructures will be examined using the continuum finite element method (FEM).

## 2 Results and Discussion

The framework of this study is based on moment tensor potentials (MTPs),^[^ ^37^ ^]^ which are a class of MLIPs that accurately describe the interatomic interactions. MTPs include parameters that are optimized by trying to reproduce the results in training datasets.^[^ ^38^ ^]^ We next discuss the creation of the ab initio training set for the passive fitting of MLIPs. To study the mechanical properties of pristine phases, AIMD calculations were performed over the rectangular supercells with only 48 atoms. Since our objective is to reproduce the complete stress–strain relations and capture the rupture, AIMD calculations were conducted over stress‐free and strained monolayers. For every structure, in order to enhance the transferability of trained MLIPs, the temperature was gradually increased from 200 to 1000 K, which is facilitating to sample stretched and highly deformed structures. We considered six strained structures, and for the sake of computational efficiency, the total time of AIMD simulations for all samples was kept to be less than 4500 time steps. Due to high correlations between the sequential AIMD configurations, only every 4th step of the full AIMD trajectories were included in the initial training set for the fitting of the preliminary MTP. Since some useful configurations in the full dataset might be removed during the subsampling scheme, the accuracy of the preliminary MTP was subsequently evaluated over the full AIMD dataset, and configurations with the highest extrapolation grades^[^ ^39^ ^]^ were selected. The identified extrapolated samples were added to the first subsampled AIMD dataset, and the final MTP was fitted. With this approach, the optimal usage of full AIMD trajectories is ensured, and the fitted MTPs are expected to show enhanced accuracy and stability.

**Figure 2:**
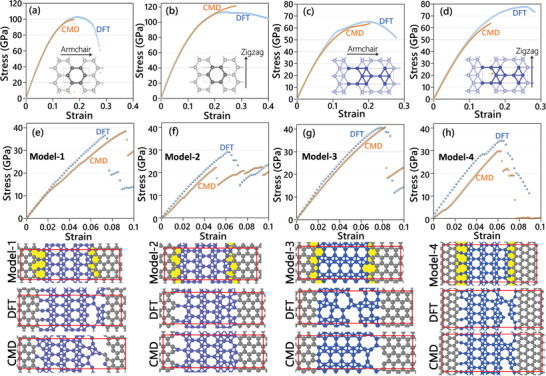
Comparison of DFT results with MTP‐based CMD at 1 K temperature for the uniaxial stress–strain response of pristine a,b) graphene and c,d) borophene and e–h) four graphene/borophene coplanar models. The red boxes show the boundary of modeled periodic cell.

In **Figure** **2**, we examine the accuracy of the trained MTPs in the evaluation of uniaxial stress–strain responses of pristine graphene and borophene monolayers. The thickness of graphene and borophene monolayers are assumed to be 3.35 and 2.9 Å,^[^ ^40^, ^41^ ^]^ respectively. The plotted stress–strain relations are uniaxial, meaning that during the tensile loading the stress along the perpendicular direction of loading is kept at negligible values. Since our goal is to compare MTP‐based CMD results with those by DFT at the ground state (0 K), in our CMD modeling the temperature is kept at 1 K. We also investigate the mechanical responses for the uniaxial loading along the armchair and zigzag directions. For both graphene and borophene, it is clear that MTP‐based CMD excellently reproduces the initial linear part of the stress–strain relation, associated with the elastic modulus. Graphene is well‐known to be a brittle membrane,^[^ ^42^, ^43^ ^]^ meaning that after reaching the ultimate tensile strength point the material is expected to abruptly crack and fail. In general, by decreasing the temperature the brittleness enhances. This phenomenon is however not reproduced by the DFT‐based results, due to the fact that the atomic vibrations are not considered. It is a highly appealing finding that MTP‐based CMD conducted at 1 K temperature not only clearly reveals the expected brittle failure in graphene but also very closely reproduces the directional dependent ultimate tensile strengths estimated by the DFT method. The elastic modulus and tensile strength of graphene by the MTP‐based CMD are predicted to be 0.99 TPa and 99–121 GPa, respectively, which are in close agreement with the experimentally measured values by Lee et. al.^[^ ^1^ ^]^ In Figure S1, Supporting Information, we also compare the phonon dispersion relations of graphene under different biaxial strain predicted by DFT and the passively fitted MTP. The presented data not only reveal high accuracy of trained MTP in describing the acoustic and optical modes in unstrained graphene but also very precisely predict the biaxial strain, where the dynamical instability occurs in graphene. As it is clear, the fitted MTP not only outperforms the classical interatomic potentials for the modeling of the mechanical response and phonon dispersion of graphene but also clearly exhibits its brittle nature, which from DFT‐based results is not realizable. The elastic modulus of χ_3_ borophene by the DFT is predicted to be 600 and 640 GPa along the zigzag and armchair directions, respectively, consistent with previous reports.^[^ ^44^, ^45^ ^]^ For the case of χ_3_ borophene, similarly to graphene, the MTP‐based CMD predicts the failure to be brittle. The tensile strength of χ_3_ borophene by the DFT is predicted to be 77.6 and 65.1 GPa along the zigzag and armchair directions, respectively. As shown in Figure 2c, using the MTP‐based CMD the tensile strength along the armchair direction is found to be 63.6 GPa, in an excellent agreement with that predicted by DFT (65.1 GPa). Along the zigzag direction, the developed classical model nonetheless underestimates the ultimate tensile strength by 18% (63.6 GPa). As it is clear, MTP‐based CMD model exhibits excellent accuracy for the graphene, but for the tensile strength of borophene with more complex chemistry, it yields a maximum of 18% disagreement with DFT estimations. It is worthwhile to note that for the case of borophene, the classical interatomic potentials cannot even reproduce the lattice parameters^[^ ^13^ ^]^ and the predicted stress–strain relations show irregular patterns and predicted tensile strengths are considerably different from DFT results.^[^ ^12^, ^13^ ^]^

Next, we shift our attention to explore the mechanical responses of coplanar graphene/borophene heterostructures. As shown in Figure 2, we constructed four different models of graphene/borophene heterostructures and optimized them using the DFT approach. Since our DFT calculations are within the plane‐wave approach and the models are periodic in all directions, every model contains two interfaces between graphene and borophene domains. From the constructed heterostructure models, we could distinguish the formation of seven different interfaces between graphene and borophene lattices. For every heterostructure model, we conducted the AIMD calculations and trained a specific MTP using the same procedure as that employed for the pristine sheets. In the developed training datasets, the AIMD trajectories for pristine lattices are also included (originally subsampled to include every 20th configuration). One specific challenge of plane‐wave DFT schemes is that due to the applied periodic boundary conditions, it is not straightforward to examine the mechanical response of a single interface. In order to examine the accuracy of the trained MTPs for the evaluation of mechanical properties, we compared the uniaxial stress–strain response of every heterostructure model by DFT at the ground state and MTP‐based CMD at 1 K temperature and the acquired results are depicted in Figure 2. Remarkable agreement for the predicted ultimate tensile strengths is noticeable for the heterostructure models of 1 and 3. The maximum discrepancy occurs for model 2, in which the MTP‐based CMD underestimated the DFT prediction by around 23%. In Figure 2, we also illustrate the failure evolution by the MTP‐based MD and DFT for every heterostructure model, which also highlights the remarkable accuracy of developed interatomic potentials in reproducing the failure mechanism. The comparisons between MTP‐based CMD and DFT results shown in Figure 2 reveals the promising accuracy of the developed classical models in describing the mechanical response of complex nanostructures. Nonetheless, the accuracy of MTP‐based CMD can be yet improved by modifying the potentials’ hyperparameters or employing active learning, which requires more expanded studies.

**Figure 3:**
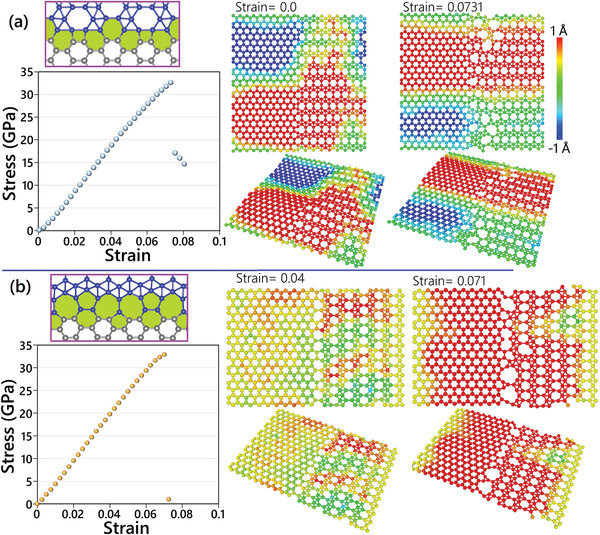
Examples of MTP‐based CMD results for the uniaxial tensile simulation of two graphene/borophene interfaces. The color coding represents the out‐of‐plane displacement at each strain level.

At this point, we are able to directly explore the mechanical response of various interfaces formed between graphene and borophene at room temperature using the MTP‐based CMD. Note that such calculations are extensively expensive to be conducted with DFT or are inaccurate/unstable with currently available empirical potentials. Seven different possible interfaces between the graphene and borophene lattices are considered. It is worthwhile to mention that in polycrystalline graphene, interfaces between different grains include mostly pentagon/heptagon dislocation pairs.^[^ ^46^ ^]^ Due to the remarkable differences in the borophene and graphene lattices, diverse interfaces can form depending on the misorientation angle and boundary atom configurations. As highlighted in Figure 2 for different heterostructure models, the interfaces between graphene and borophene can include tetragon, pentagon, hexagon, heptagon, octagon, and nonagon dislocations. We next study the mechanical properties of various interfaces by performing quasi‐static uniaxial tensile simulations. In order to minimize the effects of the loading strain rate, strain was applied with steps of 0.002 and after straining the structures were relaxed to reach negligible stress along the perpendicular direction of loading at 300 K using the NPT ensemble for 0.1 million time steps. The stresses were averaged over the last 0.05 million time steps to report the uniaxial stress–strain relations. Examples of MTP‐based CMD results for the uniaxial tensile simulation of two graphene/borophene interfaces are illustrated in **Figure** **3**. The results for considered interfaces are elaborately shown in Supporting Information (find Figure S2–S8, Supporting Information, and Supplementary Videos). Because of different lattice constants of graphene and borophene, noticeable buckling and deflection occur for the unstrained samples. Despite dissimilar dislocation configurations for different interfaces, our results for the ultimate tensile strength points are found to be very close and vary between 25–33 GPa. Moreover, our first‐principles‐based results reveal that in all cases the initial damage in the heterostructure initiates from the dislocation cores and subsequently extends throughout the interface. In all cases, the graphene regions owing to their distinctly higher tensile strengths remain completely intact, while borophene lattices near the interface undergo remarkable distortions. Some interfaces show a ductile failure mechanism, for which strong C‐B‐B‐ or B‐B‐ chains form that tend to keep the two sides connected during the deformation after the initial rupture (see Supplementary Videos). These observations reveal that dissimilar lattices and mechanical characteristics of graphene and borophene are the dominant factors and the form of dislocation cores at the interface does not substantially affect the ultimate tensile strength. Therefore, although a more extensive ensemble of interfaces could be constructed between graphene and borophene lattices, one expects close mechanical characteristics as those we presented.

From the multiscale point of view, for the modeling of graphene/borophene heterostructures, apart from the microstructure, one needs to know the mechanical properties of pristine phases and their interaction characteristics. In the previous step, we evaluated the mechanical response of different graphene/borophene interfaces at room temperature. In this work and as a common approach, the contact interaction properties between graphene and borophene lattices are defined using the cohesive zone elements, which is an effective way to simulate the deboning and damage evolution in composite structures. The acquired uniaxial stress–strain relations for different interfaces were converted to the traction–separation relations in order to define the cohesive zone‐contact properties. As discussed earlier, for few cases, the stress after the ultimate tensile strength does not sharply drop to zero due to the formation of atomic chains. In the definition of cohesive zone properties, we nonetheless assume sharp drops after reaching the ultimate tensile strength. This can be a realistic assumption, taking into account that CMD‐based calculations are yet conducted at very short time periods, and this might affect the deformation after the initial rupture. We also evaluated the mechanical properties of graphene and borophene at room temperature using the quasi‐static uniaxial tensile simulations, and the results are shown in Figure S9, Supporting Information. Herein we also analyzed the strain rate effect, which reveals that the curves obtained for different strain rates coincide, and only the maximum tensile strength point changes. For the case of graphene for both considered loading directions, it is noticeable that with the strain rate of 10^9^ s^−1^ the results are convincingly converged. For the case of borophene, despite quasi‐static loading, a slightly decreasing trend is yet observable. We note that for the CMD modeling at 1 K temperature, the strain rate effect is found to be completely negligible. For the sake of simplicity in our highly non‐linear continuum modeling, we assume isotropic mechanical responses for graphene and borophene pristine phases. As a safe approach (lower‐bound), we utilized the predicted mechanical properties for the direction with the lowest values, armchair and zigzag for graphene and borophene, respectively (see Figure S9, Supporting Information).

**Figure 4:**
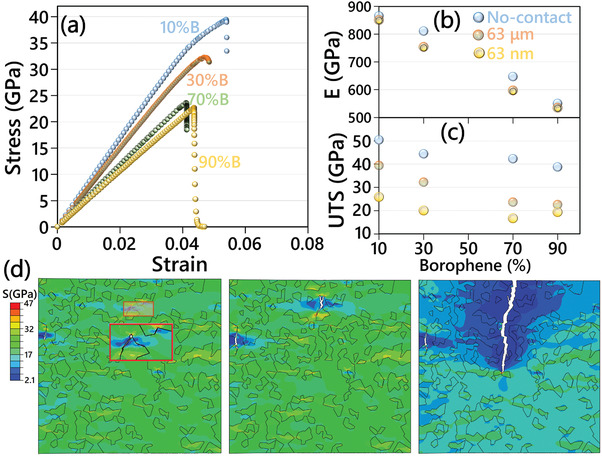
a) First‐principles multiscale modeling results for the stress–strain responses of graphene/borophene heterostructure with domain size of 63 µm. b) Elastic modulus and c) ultimate tensile strength of heterostructure as a function of borophene content. Two domain sizes of 63 nm and 63 µm are considered and the results are compared with those of perfect bonding (no contact). d) Deformation of a heterostructure with domain size of 63 µm and 70% borophene content. Contours present the von‐Mises stress contours.

After evaluating the mechanical properties of pristine phases and their interfaces using the first‐principles CMD method, we are now able to conduct the continuum modeling. As illustrated in Figure S10, Supporting Information, we develop polycrystalline specimens with 1000 individual grains on the basis of Voronoi method in order to construct heterostructure samples. According to the volume concentration of each phase, every grain is randomly assigned to be either graphene or borophene by defining their associated stress–strain relations. The grains with similar materials were then merged, that is, no interfaces were assumed between them. The predicted cohesive zone properties were then randomly assigned to describe the mechanical bonding between dissimilar crystals. In order to explore the size effect, we considered three different systems, two models with equivalent domain sizes of 63 nm and 63 µm, and one model with perfect bonding between graphene and borophene phases (with no cohesive zone elements). The damage in pristine phases is defined to occur as the maximum tensile strength is reached. By applying strains on one side of the model, the deformation is simulated and stress values were recorded. In **Figure** **4**a, the predicted stress–strain relations for the mechanical response of heterostructures with the domain size of 63 µm are compared. As expected with increasing the content of borophene, the elastic modulus, and ultimate tensile strength decrease. In Figure 4b,c, we compare the predicted elastic modulus (E) and ultimate tensile strength (UTS) as a function of borophene phase content for the three considered systems. As a general finding, by increasing the borophene content and decreasing the domain size, the elastic modulus and ultimate tensile strength decrease considerably and slightly, respectively. It is conspicuous that the different models predict very close elastic modulus for the constructed heterostructures, particularly for those with borophene contents of 10 and 90%. The differences, however, increase when the borophene content is either 30% or 70%. We simply remind that by increasing the volume content from 10% to 30%, more interfaces form in the system, and such that the importance of contact definition increases. Our results presented here clearly show that for the analysis of elastic modulus, the definition of interaction properties yields negligible effects. In contrast, for the case of ultimate tensile strength, different models predict noticeably different values. More importantly, the models without cohesive zone elements considerably overestimate the ultimate tensile strength. By decreasing the domain size, more interfaces form in per unit area, and since the strength of these interfaces is lower than the native phases, the effective ultimate strength of the heterostructure decrease. An interesting phenomenon is observable for the model with a domain size of 63 nm, for which the sample with 90% content of borophene shows slightly higher tensile strength than the one with 70%. As discussed earlier, the aforementioned sample with a higher borophene content includes fewer interfaces, and despite its lower percentage of ultra‐strong graphene, it can yield higher ultimate tensile strength. As an example, the deformation of a heterostructure with a domain size of 63 µm and 70% borophene content is illustrated in Figure 4d (see Supplementary Videos). It is clear that the preliminary failures initiate by the deboning in a few interfaces. The crack in the system grows by coalescences between two or more close debonded interfaces, which rapidly extend through the borophene phase and preferably alongside neighboring interfaces. We found that when the crack tip faces the graphene grains, it can even pass by breaking the graphene lattice (see Supplementary Videos). In accordance with our results, the crack growth and damage in composite and heterostructure materials have stochastic nature and the configuration and coalescences of original debondings define the ultimate tensile strength, and therefore for different heterostructures with the same domain size and volume contents the tensile strength may change. Therefore, while the elastic properties are dominated by the content of every phase, the predicted ultimate tensile strength change also depending on the domain size, size of heterostructure models, and different configurations and geometries for grains.

## 3 Conclusion

In summary, we develop the concept of first‐principles multiscale modeling of mechanical properties, where MLIPs enable the efficient bridging of ab initio modeling to continuum scale. The proposed hierarchy starts from DFT calculations for preparing the dataset and subsequent fitting of MLIPs. It is followed by CMDs simulations to explore the complex mechanical behavior at room temperature and finally empower the continuum finite element modeling of mechanical response at the macroscopic scale. We employed the proposed approach to examine the mechanical/failure response of important but unexplored graphene/borophene coplanar heterostructures. Our results reveal that machine‐learning potentials outperform the common classical models for the modeling of mechanical properties of pristine graphene and borophene. More importantly, we could accurately investigate the mechanical response of complex interfaces, for which there exist no accurate or computationally feasible classical or ab initio based methods. Our study highlights that MLIPs offer extraordinary capabilities to marry the first‐principles accuracy with multiscale modeling and thus enable the modeling of complex nanostructures at continuum level with ab initio level accuracy without paying unaffordable computational costs. The proposed approach shows outstanding and robust potential to develop fully automated platforms, to design, optimize and explore thermal, mechanical and failure responses of materials and structures at continuum level by capturing atomistic effects, and with inherent precision of first‐principles calculations.

## 4 Experimental Section

DFT calculations were carried out using the *Vienna Ab initio Simulation Package* (VASP)^[^ ^47^, ^48^, ^49^ ^]^ with generalized gradient approximation and Perdew–Burke–Ernzerhof^[^ ^50^ ^]^ functional with a plane‐wave cutoff energy of 500 eV. For geometry optimizations, atoms and lattices were relaxed according to the Hellman–Feynman forces using the conjugate gradient algorithm until atomic forces drop to lower than 0.01 eV Å^−1^. Mechanical properties were examined by conducting uniaxial tensile simulations. In these calculations, the stresses along the two perpendicular loading directions should stay negligible during various loading stages. Due to the contact with vacuum along the normal direction of the monolayers, the stress along this direction automatically reaches a negligible value upon the geometry optimization. For the other planar direction, the periodic box size was altered to ensure that the corresponding stress was negligible. For the graphene and borophene pristine phases, rectangular unitcells with 13 × 13 × 1 Monkhorst–Pack^[^ ^51^ ^]^ k‐point grid were considered. For the tensile simulations of heterostructures, a coarse grid of 2 × 2 × 1 was employed. Datasets were prepared using the AMDSs under canonical ensemble algorithm of Nosé^[^ ^52^ ^]^ and time step of 1 fs using a 2 × 2 × 1 k‐point gird. Datasets were prepared using the AIMDs under canonical ensemble algorithm of Nosé^[^ ^52^ ^]^ and time step of 1 fs using a 2 × 2 × 1 k‐point gird. MTPs with 329 parameters for pristine graphene and borophene, and 449 parameters for heterostructures were trained using the MLIP package.^[^ ^38^ ^]^ Phonon dispersions were obtained by density functional perturbation theory simulations over 6 × 6 × 1 supercells with a 3 × 3 × 1 k‐point grid using the PHONOPY code.^[^ ^53^ ^]^ Phonon dispersions were also calculated using the MTPs and PHONOPY as explained in the earlier study.^[^ ^54^ ^]^ OVITO^[^ ^55^ ^]^ package was employed to plot the atomistic results.

CMDs simulations were conducted using the LAMMPS^[^ ^56^ ^]^ package. In accordance with first‐principles modeling, mechanical responses were evaluated by conducting the uniaxial tensile simulations with a time increment of 0.5 fs. Before applying the loading conditions, all structures were equilibrated using the Nosé–Hoover barostat and thermostat method (NPT). For the satisfaction of uniaxial loading condition, a constant engineering strain after applying the loading strain, NPT method was employed to control the temperature fluctuations and also adapt the box size along the perpendicular direction of loading to reach negligible tensile stress. Continuum models of heterostructures were constructed and simulated using the ABAQUS/Standard package along with python scripting. Ductile damage and cohesive zone elements were defined to simulate the failures in pristine phases and interfaces, respectively.

## Conflict of Interest

The authors declare no conflict of interest.

## Supporting information

Supporting Information

Supplemental Video 1

Supplemental Video 2

Supplemental Video 3

Supplemental Video 4

Supplemental Video 5

Supplemental Video 6

## Data Availability

Research data are available via: https://doi.org/10.17632/yrn7p7w37f.1.
