# Supplementary material for: First‐Principles Multiscale Modeling of Mechanical Properties in Graphene/Borophene Heterostructures Empowered by Machine‐Learning Interatomic Potentials
Source: Adv Mater. 2021 Jul 23;33(35):2102807. doi: 10.1002/adma.202102807 (PMC11469135; doi:10.1002/adma.202102807)
Supplement: Supplementary file 1 — Supporting Information [file ADMA-33-2102807-s004.pdf]

© 2021 Wiley-VCH GmbH

ADVANCED MATERIALS

Supporting Information

for *Adv. Mater.,* DOI: 10.1002/adma.202102807

First-Principles Multiscale Modeling of Mechanical Properties in Graphene/Borophene Heterostructures Empowered by Machine-Learning Interatomic Potentials

Bohayra Mortazavi, Mohammad Silani, Evgeny V. Podryabinkin, Timon Rabczuk, Xiaoying Zhuang,* and Alexander V. Shapeev*

Supplementary Information

First-Principles Multiscale Modeling of Mechanical Properties in Graphene/Borophene Heterostructures Empowered by Machine-Learning Interatomic Potentials

Bohayra Mortazavi*[a,b], Mohammad Silani[c], Evgeny V. Podryabinkin[d],

Timon Rabczuk[e], Alexander V. Shapeev[d] and Xiaoying Zhuang[a,b]

[a]Chair of Computational Science and Simulation Technology, Institute of Photonics, Department of Mathematics and Physics, Leibniz Universität Hannover, Appelstraße 11,30167 Hannover, Germany.
[b]Cluster of Excellence PhoenixD (Photonics, Optics, and Engineering–Innovation Across Disciplines), Gottfried Wilhelm Leibniz Universität Hannover, Hannover, Germany.
[c]Department of Mechanical Engineering, Isfahan University of Technology, Isfahan, 84156-83111, Iran
[d]Skolkovo Institute of Science and Technology, Skolkovo Innovation Center, Nobel St. 3, Moscow 143026, Russia.
[e]College of Civil Engineering, Department of Geotechnical Engineering, Tongji University, Shanghai, China.

*bohayra.mortazavi@gmail.com

Computational details are accessible via: http://dx.doi.org/10.17632/yrn7p7w37f.1
Which contains:
"Important Notes.pdf" which contains important information for straightforward training of MTPs and details of various folders.
"LAMMPS-Inputs.zip" folder includes: four examples of LAMMPS input scripts to study the mechanical properties at 300 K with the MTPs interatomic potentials.
"AIMD-Inputs.zip" folder includes: VASP input parameters for the AIMD simulations.
"POSCARs-for-AIMD.zip" folder includes: all considered structures for AIMD calculations.
"Heterostructure-Models.zip" folder includes: constructed four graphene/borophene heterostructure models.
"Training-Data-Full.zip" folder includes: full obtained AIMD trajectories.
"Clean-MTP.zip" folder includes: untrained MTPs.
"FEM-ABAQUS-Models.zip" folder includes: examples of ABAQUS input files for two heterostructures with the domain size of 63 µm (ABAQUS version 6.20).

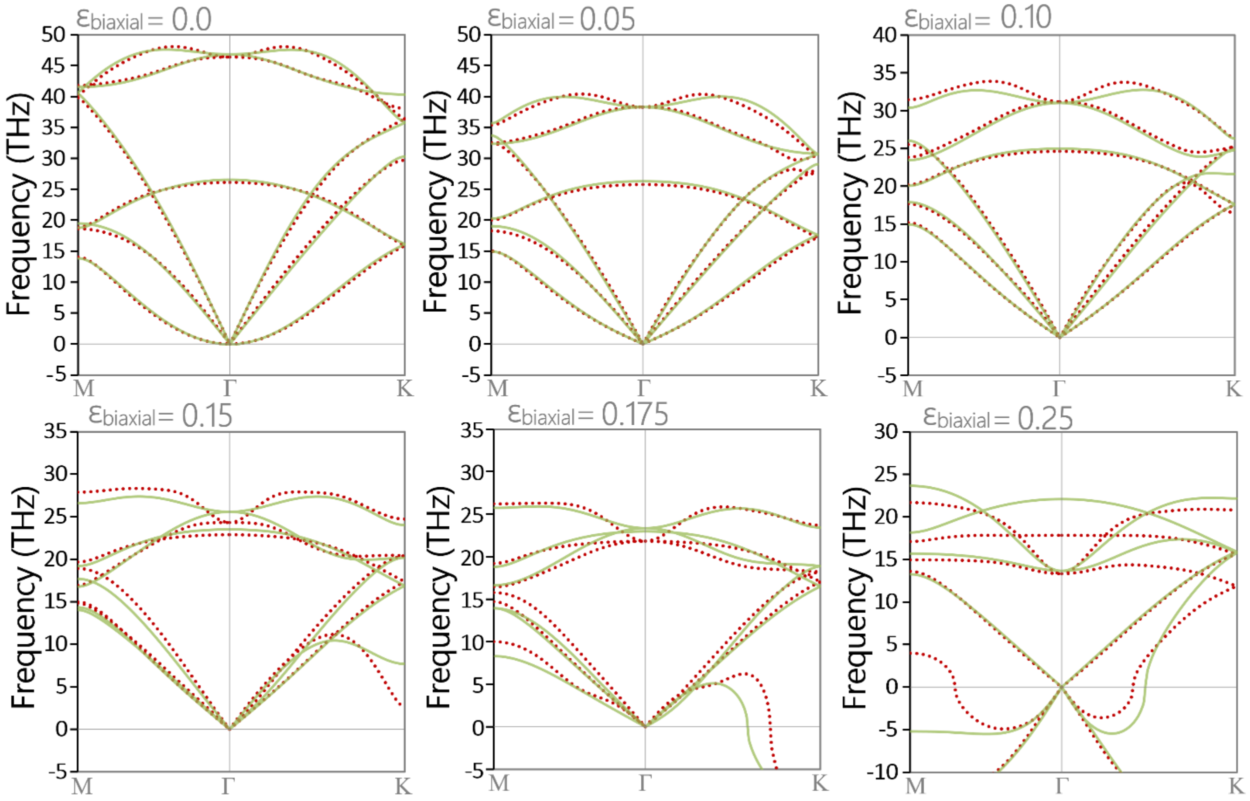

Fig. S1, Phonon dispersion relations of graphene by DFPT (red-dotted lines) method and trained MTP (continuous green lines) under different biaxial strains ($\varepsilon_{biaxial}$).

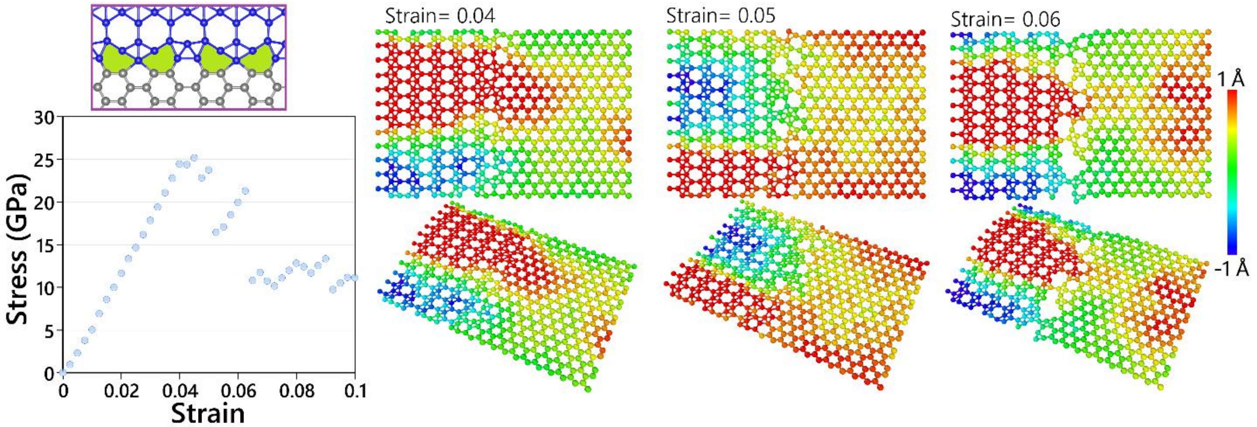

Fig. S2, MTP-based CMD results for the uniaxial stress-strain response and deformation of a heterostructure with the illustrated interface at room temperature. The color coding represents the out-of-plane displacement at each strain level.

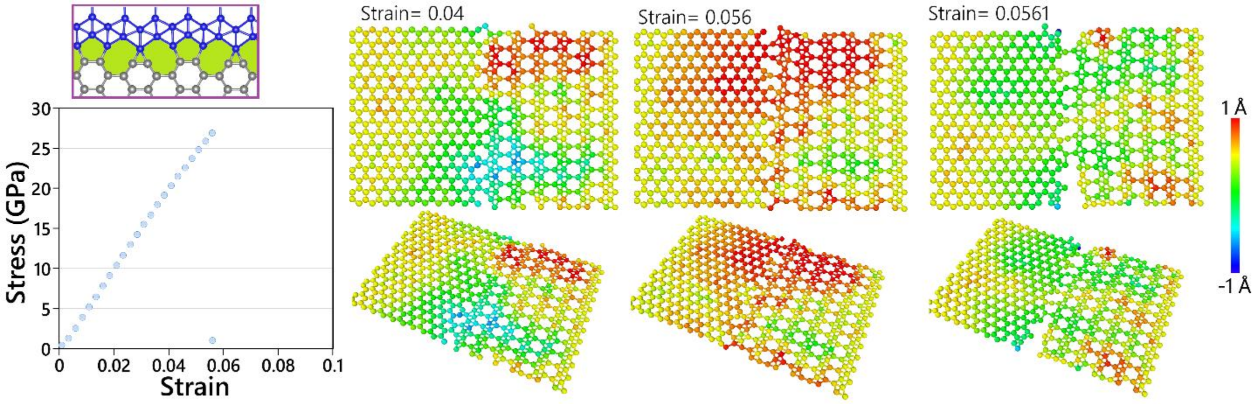

Fig. S3, MTP-based CMD results for the uniaxial stress-strain response and deformation of a heterostructure with the illustrated interface at room temperature. The color coding represents the out-of-plane displacement at each strain level.

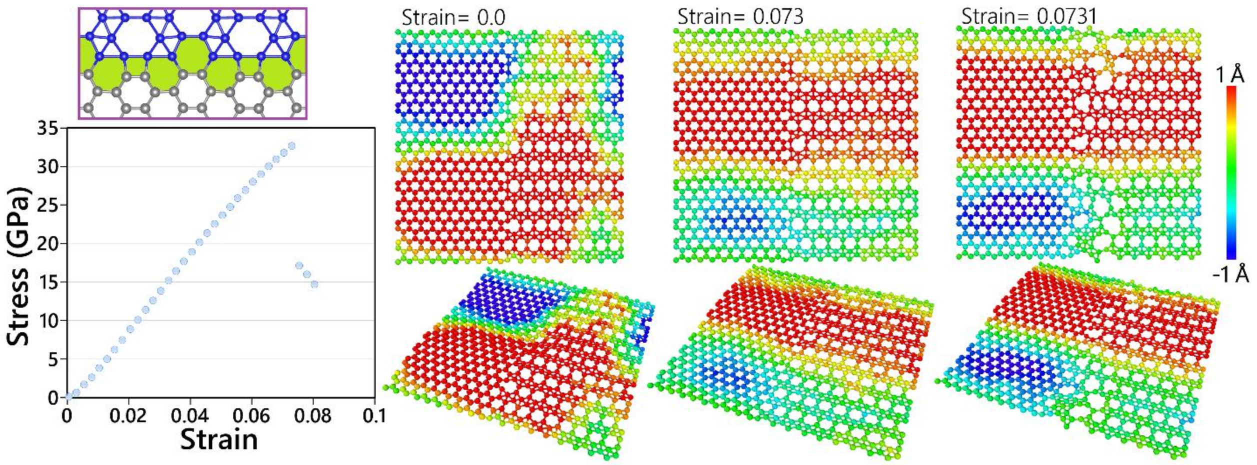

Fig. S4, MTP-based CMD results for the uniaxial stress-strain response and deformation of a heterostructure with the illustrated interface at room temperature. The color coding represents the out-of-plane displacement at each strain level.

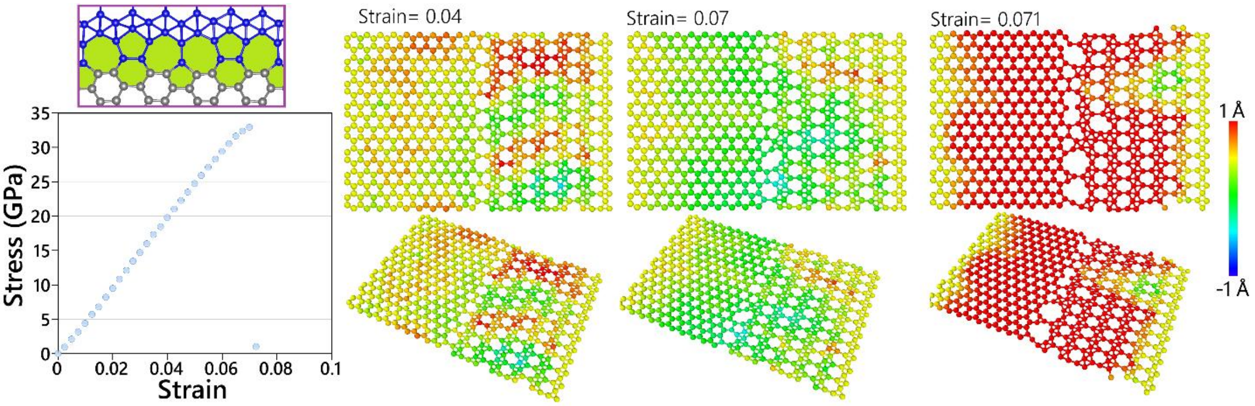

Fig. S5, MTP-based CMD results for the uniaxial stress-strain response and deformation of a heterostructure with the illustrated interface at room temperature. The color coding represents the out-of-plane displacement at each strain level.

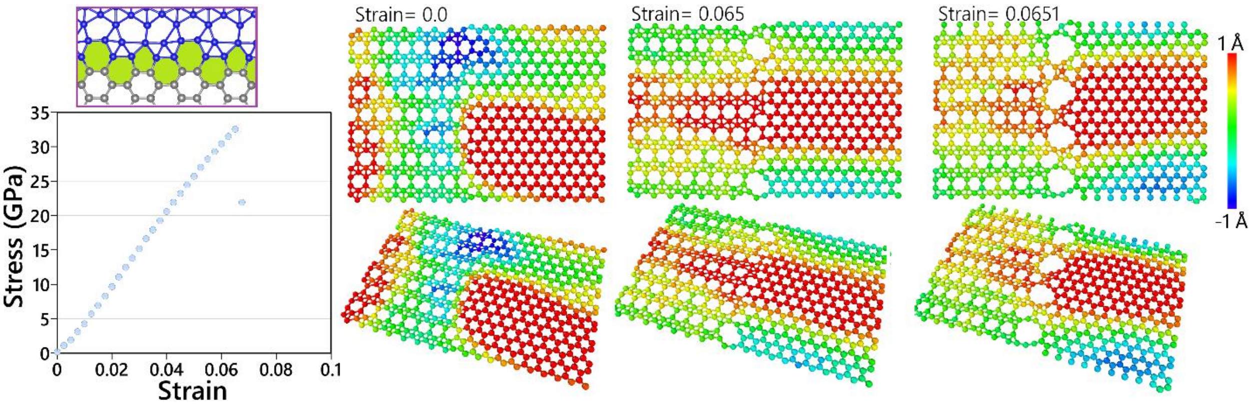

Fig. S6, MTP-based CMD results for the uniaxial stress-strain response and deformation of a heterostructure with the illustrated interface at room temperature. The color coding represents the out-of-plane displacement at each strain level.

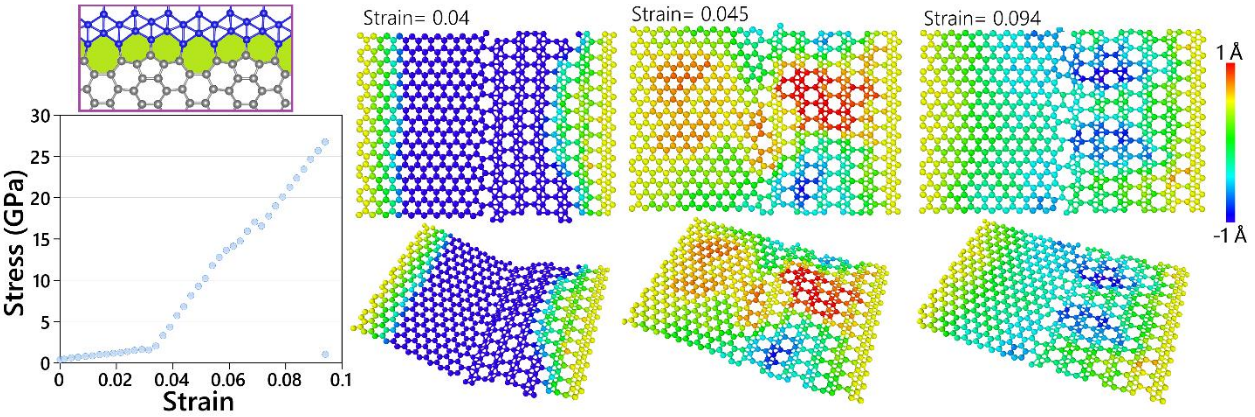

Fig. S7, MTP-based CMD results for the uniaxial stress-strain response and deformation of a heterostructure with the illustrated interface at room temperature. The color coding represents the out-of-plane displacement at each strain level.

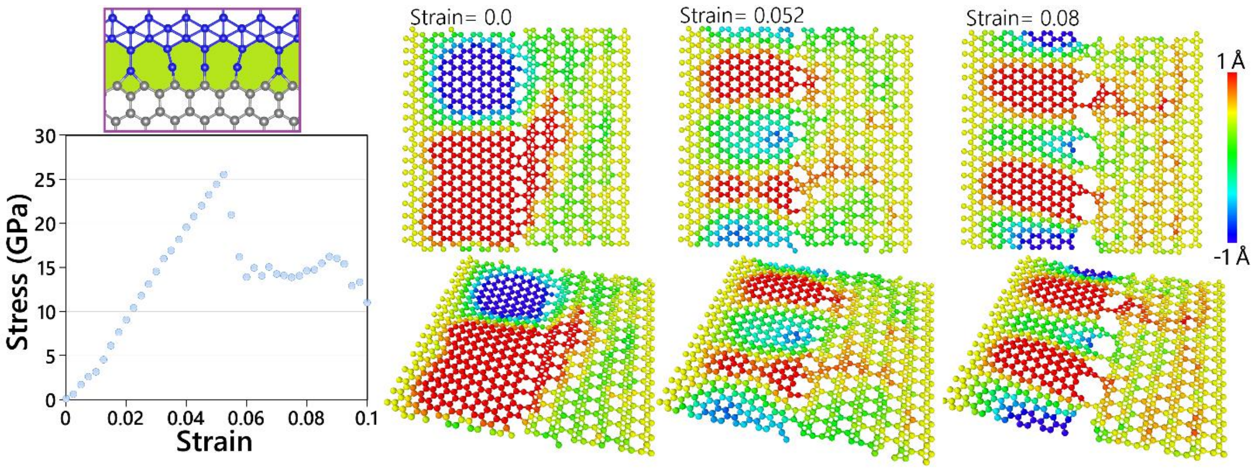

Fig. S8, MTP-based CMD results for the uniaxial stress-strain response and deformation of a heterostructure with the illustrated interface at room temperature. The color coding represents the out-of-plane displacement at each strain level.

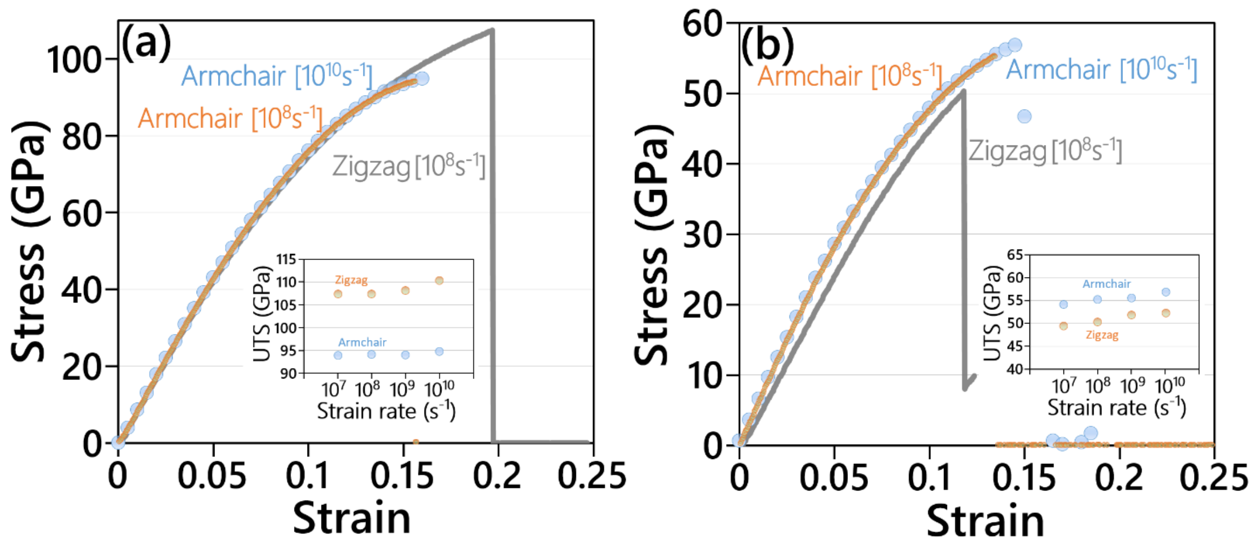

Fig. S9, MTP-based CMD results for the uniaxial stress-strain response of pristine graphene and borophene at room temperature at different [strain rates].

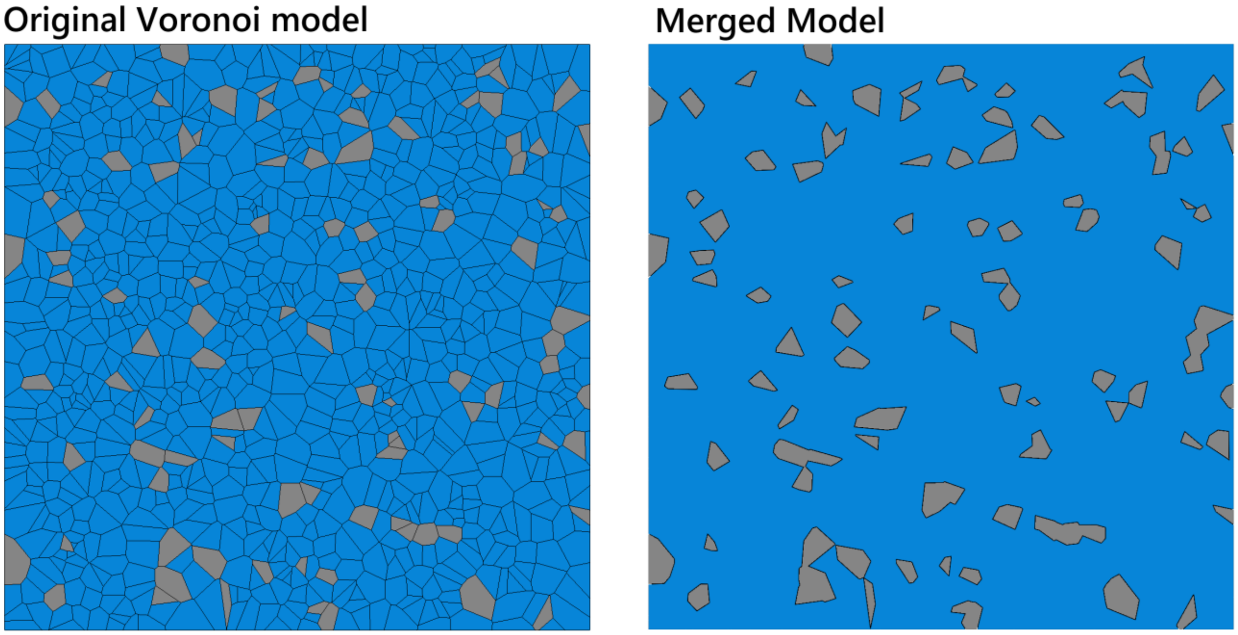

Fig. S10, Construction of Voronoi based polycrystalline models of heterostructures in ABAQUS/standard.
